# Imaging of tuberous sclerosis complex: a pictorial review

**DOI:** 10.1590/0100-3984.2016.0020

**Published:** 2017

**Authors:** Felipe Mussi von Ranke, Igor Murad Faria, Gláucia Zanetti, Bruno Hochhegger, Arthur Soares Souza Jr., Edson Marchiori

**Affiliations:** 1 MD, Universidade Federal do Rio de Janeiro (UFRJ), Rio de Janeiro, RJ, Brazil.; 2 MD, PhD, Universidade Federal do Rio de Janeiro (UFRJ), Rio de Janeiro, RJ, Brazil.; 3 MD, PhD, Santa Casa de Porto Alegre, Porto Alegre, RS, Brazil.; 4 MD, PhD, Faculdade de Medicina de São José do Rio Preto (Famerp) and Ultra X, São José do Rio Preto, SP, Brazil.

**Keywords:** Tuberous sclerosis, Computed tomography, Magnetic resonance imaging

## Abstract

Tuberous sclerosis complex (TSC) is a genetically determined hamartomatous neurocutaneous disease with high phenotypic variability. TSC is characterized by widespread hamartomas and benign, or rarely malignant, neoplasms distributed in several organs throughout the body, especially in the brain, skin, retina, kidney, heart, and lung. Common manifestations include cortical tubers, subependymal nodules, white matter abnormalities, retinal abnormalities, cardiac rhabdomyoma, lymphangioleiomyomatosis, renal angiomyolipoma, and skin lesions. The wide range of organs affected by the disease implies that TSC1 and TSC2 genes play important roles in the regulation of cell proliferation and differentiation. Although recent advances in treatment have improved morbidity, the prognosis remains quite poor and nearly 40% of patients die by the age of 35 years. Imaging is important in the evaluation of TSC because of its role not only in presumptive diagnosis, but also in defining the full extent of involvement. This information allows a better understanding of the behavioural phenotype, as related to lesion location. Imaging also contributes to treatment planning. This pictorial review describes common and uncommon imaging manifestations of TSC.

## Abstract (pt)

Complexo esclerose tuberosa (CET) é uma síndrome hamartomatosa neurocutânea geneticamente determinada, com elevada variabilidade fenotípica. O CET é caracterizado por hamartomas generalizados e pela presença de neoplasias benignas, ou raramente malignas, acometendo vários órgãos, especialmente cérebro, pele, retina, rins, coração e pulmões. As manifestações mais comuns incluem tubérculos corticais, nódulos subependimais, anormalidades na substância branca, anormalidades da retina, rabdomioma cardíaco, linfangioleiomiomatose, angiomiolipoma renal e lesões de pele. A ampla gama de órgãos afetados sugere que genes TSC1 e TSC2 desempenham papel importante na regulação da proliferação e diferenciação celular. Apesar de os recentes avanços no tratamento terem melhorado a morbidade, o prognóstico permanece bastante ruim e cerca de 40% dos pacientes morrem próximo aos 35 anos de idade. Estudos por imagem são importantes na avaliação do CET, em razão do seu papel não só no diagnóstico presuntivo, mas também na definição da extensão total da doença. Estas informações permitem uma melhor compreensão do fenótipo comportamental, relacionado com a localização das lesões. A imagem auxilia, também, no planejamento do tratamento.. Este ensaio pictórico descreve as manifestações de imagem comuns e incomuns do CET.

## INTRODUCTION

Tuberous sclerosis complex (TSC) is a genetically determined multisystem hamartomatous neurocutaneous disease. The organs most commonly involved are the brain, skin, kidney, lung, retina, and heart^(1)^. The wide range of organs affected by the disease implies that TSC1 and TSC2 genes play important roles in the regulation of cell proliferation and differentiation^(2)^. The classic triad of manifestations consists of facial adenoma sebaceum, epilepsy, and mental retardation^(3)^. The presence of common manifestations, including cortical tubers or subependymal nodules, white matter (WM) abnormalities, retinal abnormalities, cardiac rhabdomyoma, lymphangioleiomyomatosis (LAM), and renal angiomyolipoma (AML), allows confirmation of the diagnosis, especially when associated with skin lesions^(4)^. Imaging is important in the evaluation of TSC because of its roles not only in presumptive diagnosis, but in defining the full extent of involvement. Imaging also contributes to treatment planning.

## DIAGNOSIS

The demonstration of a pathogenic mutation in the TSC1 or TSC2 gene in normal tissue is now considered sufficient for the diagnosis of TSC, independent of clinical manifestations^(5)^. Clinical diagnostic criteria are important, however, because genetic testing may not identify a mutation in up to 25% of patients. The clinical criteria are divided into major and minor features, with definitive diagnosis defined by the presence of at least two major features or one major and two minor features. The diagnosis of TSC is considered possible in the presence of one major or two or more minor features^(5)^.

## INTRACRANIAL MANIFESTATIONS

### Cortical tubers

The expansive lesions of the central nervous system alone are not diagnostic of TSC and may be seen in other pathological conditions^(6-10)^.

**Figure 1:**
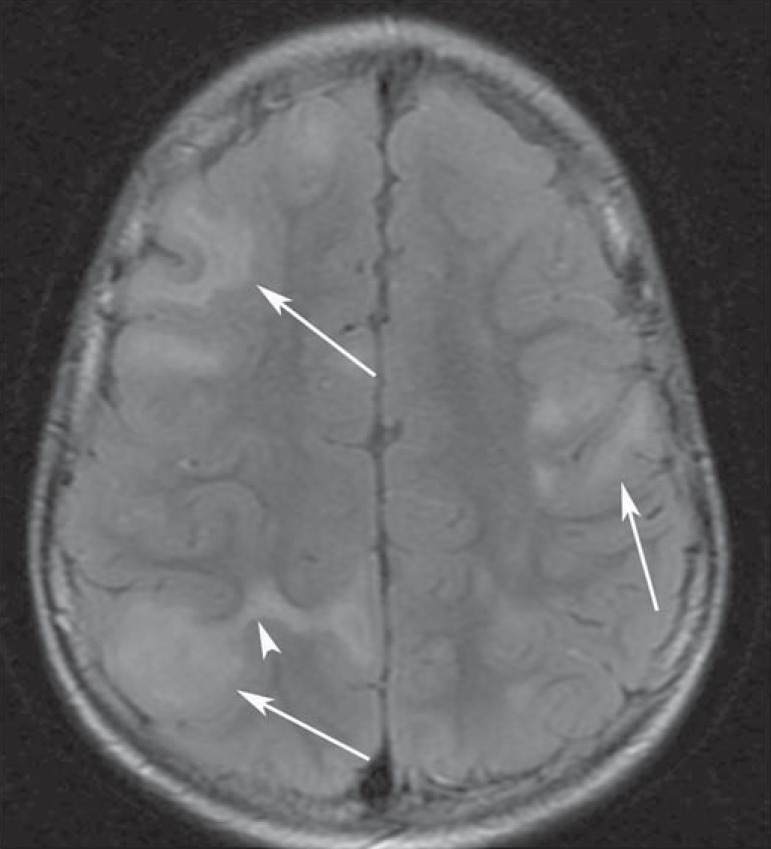
An 8-year-old boy with tuberous sclerosis complex. Axial fluid-attenuation inversion-recovery image demonstrates cortical tubers (arrows) as well-circumscribed areas of high signal intensity. Superficial white matter abnormalities are seen as hyperintense areas in relation to tubers (arrowhead).

**Figure 2:**
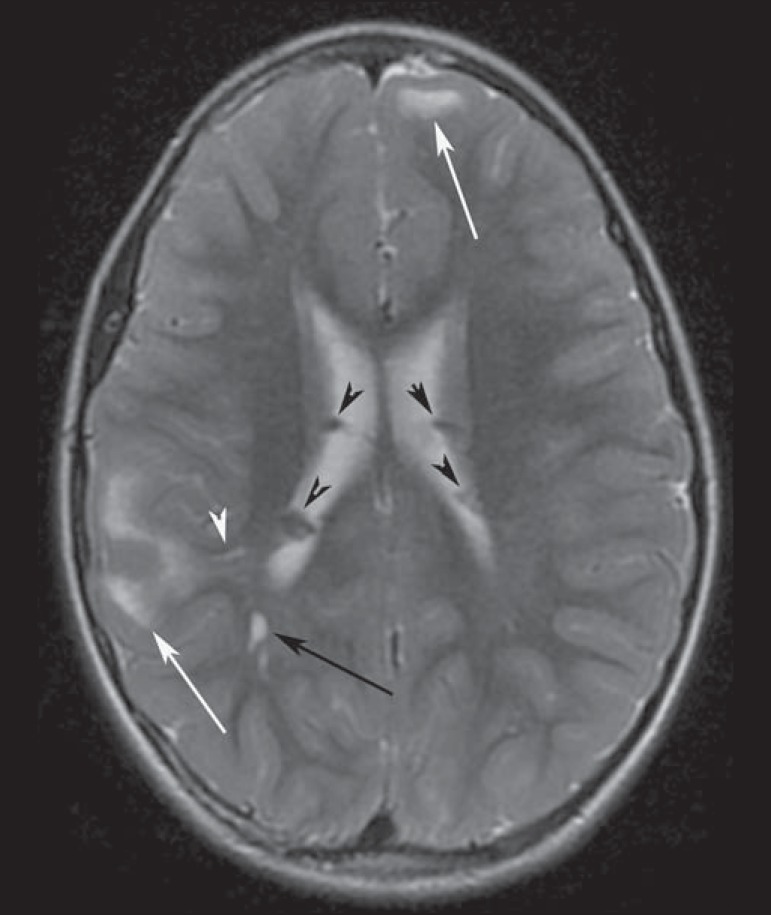
A 12-year-old boy with tuberous sclerosis complex. Axial T2-weighted image shows cortical tubers as well-circumscribed areas of high signal intensity (white arrows) and subependymal nodules along the ventricular surface (black arrowheads). Note radial migration line appearing as a thin, straight band of hyperintensity extending from the juxtaventricular white matter to the cortex (black arrow), and white matter cyst-like lesion located in deep white matter near the atrium of the right lateral ventricle (white arrowhead).

Cortical tubers are benign hamartomas detectable in the cerebral cortex in approximately 95% of patients with TSC^(4)^. They are considered to be related closely to the neurological manifestations of TSC, including epilepsy, cognitive disability, and neurobehavioural abnormalities^(4)^. On magnetic resonance imaging (MRI), cortical tubers typically appear as well-circumscribed areas of low signal intensity on T1-weighted and high signal intensity on T2-weighted sequences^(11)^ (Figures 1 and 2).

### Subependymal nodules

Subependymal nodules are common brain lesions found in patients with TSC, which represent hamartomatous growths. Typically benign, subependymal nodules can degenerate into subependymal giant cell astrocytomas (SEGAs)^(11)^. Computed tomography (CT) is useful for the detection of subependymal nodules, as they are associated with calcification far more commonly (88%) than are cortical tubers^(4)^. On MRI, subependymal nodules show intermediate signal intensity on T1-weighted images and isointense to hyperintense signals on T2-weighted images^(3,4)^ (Figure 2).

### Subependymal giant cell astrocytomas

**Figure 3:**
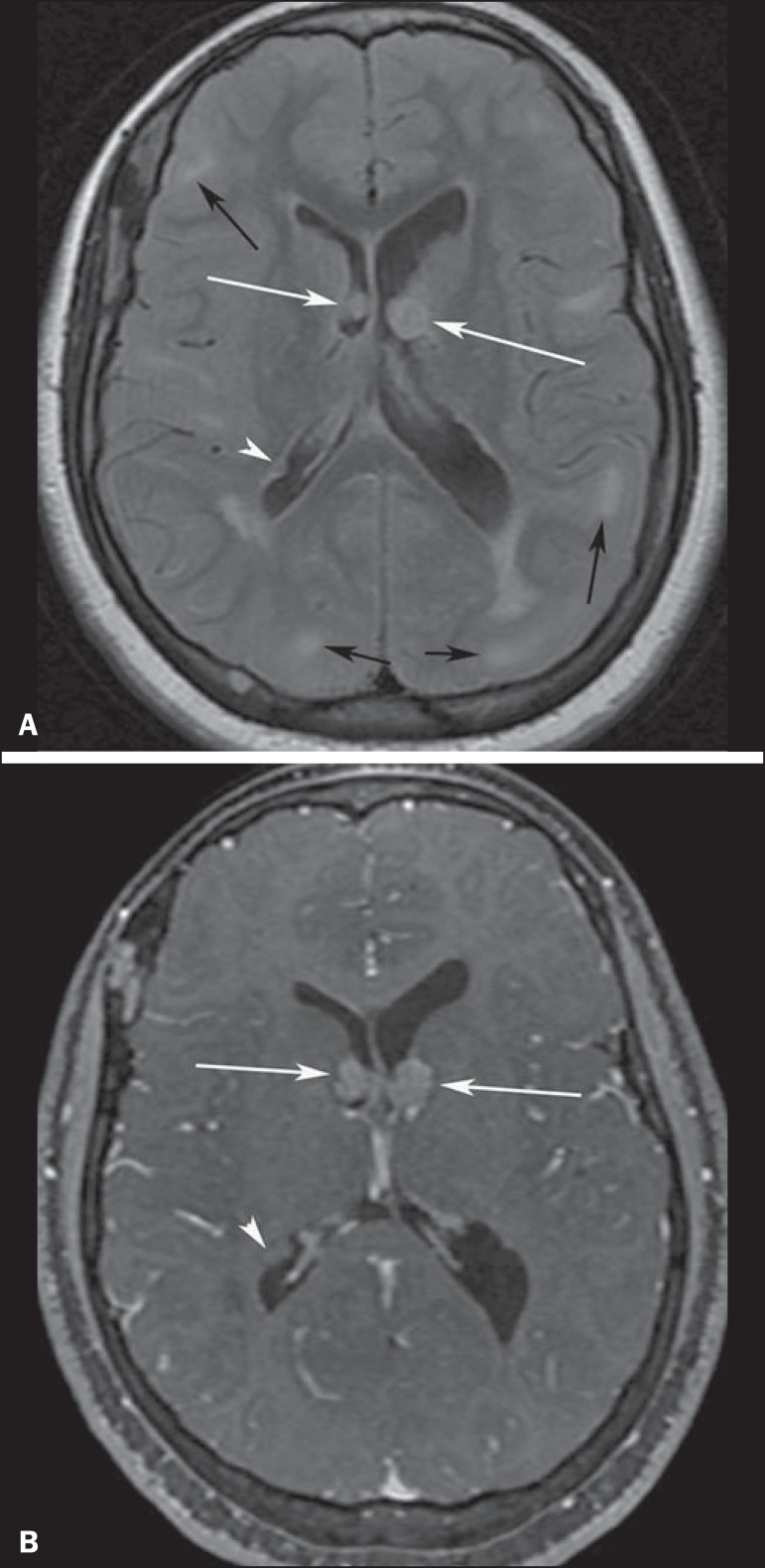
A 23-year-old woman with tuberous sclerosis complex and subependymal giant cell astrocytomas. Axial fluid-attenuation inversion-recovery image (**A**) shows heterogeneous hyperintense masses (subependymal giant cell astrocytomas) located at the frontal horn of the lateral ventricles, near the foramen of Monro (white arrows). Axial contrast-enhanced T1-weighted image (**B**) shows strong and heterogeneous enhancement of the lesions (white arrows). Note also cortical tubers (black arrows), and subependymal nodule along the right lateral ventricular surface (arrowheads), best depicted on fluid-attenuation inversion-recovery image.

SEGAs are widely accepted to be derived from subependymal nodules^(2)^. The prognosis is generally good, as SEGAs grow slowly. However, SEGA growth may result in ventricular obstruction and hydrocephalus^(4)^. SEGAs are hypo- to isointense compared with cortex on T1-weighted images and heterogeneously iso- to hyperintense on T2-weighted images^(3)^ (Figure 3).

### White matter abnormalities

WM abnormalities in TSC include superficial abnormalities associated with cortical tubers, radial migration lines (RMLs), and cyst-like WM lesions. On MRI, superficial WM abnormalities are seen as hyperintense areas on T2-weighted images and hypointense areas on T1-weighted images (Figure 1). RMLs appear as thin, straight or curvilinear bands of hyperintensity on T2-weighted images and show iso- to hypointensity on T1-weighted sequences. Cyst-like WM lesions are small well-demarcated lesions with intensity similar to that of cerebrospinal fluid. They are seen in deep WM, typically near the lateral ventricles^(4,11)^.

## PULMONARY MANIFESTATIONS

### Lymphangioleiomyomatosis

**Figure 4:**
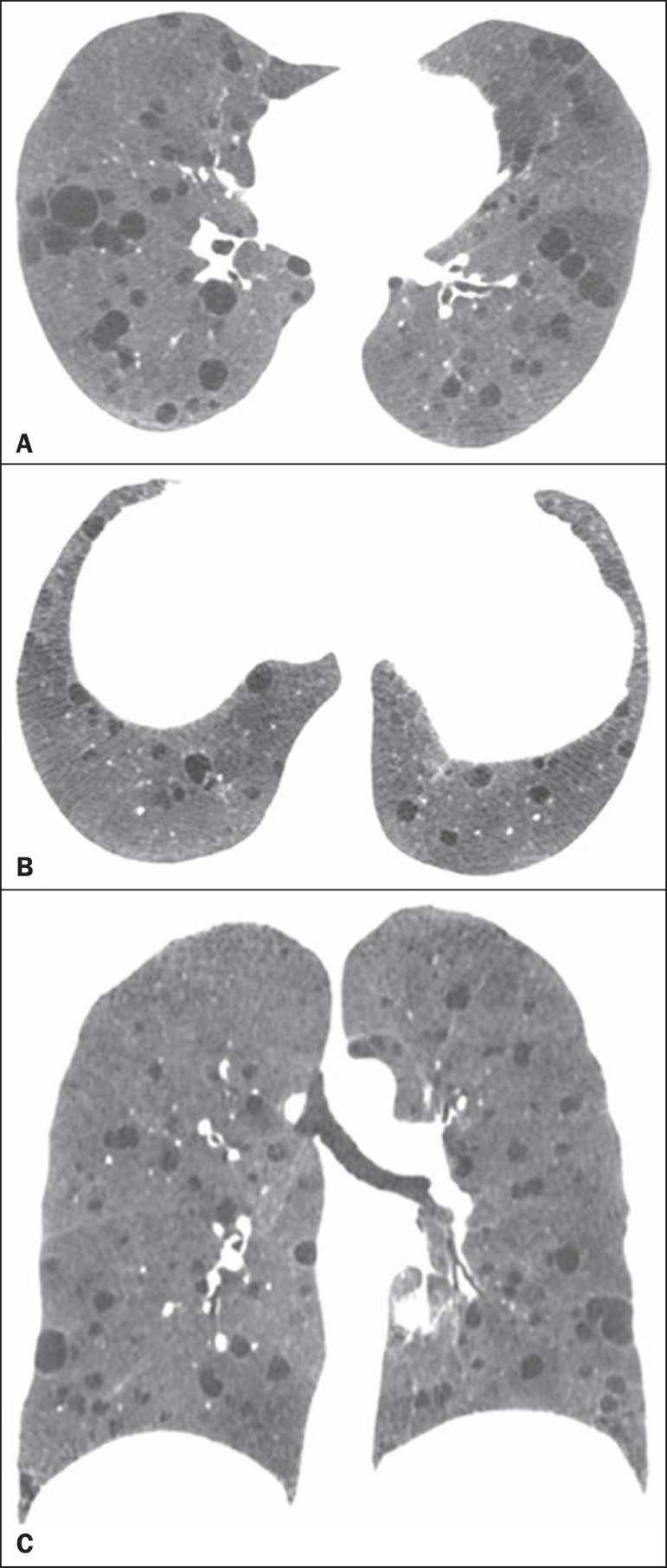
A 52-year-old woman with tuberous sclerosis complex. Axial (**A,B**) and coronal (**C**) CT mini-maximum-intensity projection images show numerous bilateral, variably sized thin-walled cysts, compatible with lymphangioleiomyomatosis.

**Figure 5:**
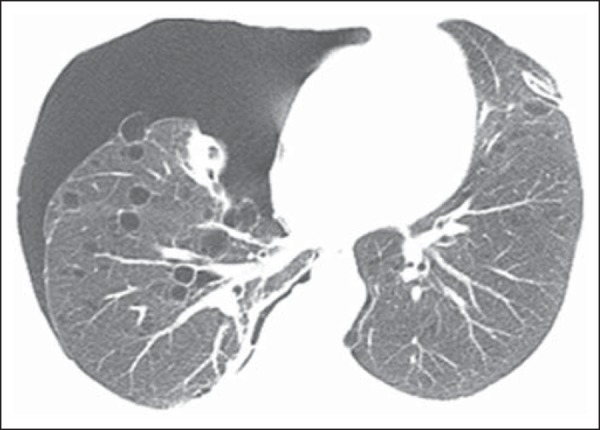
A 52-year-old woman with tuberous sclerosis complex presenting with sudden onset of right chest pain and dyspnoea. Axial CT image obtained with the lung window setting shows right-sided pneumothorax and numerous thin-walled cysts scattered throughout the lungs, compatible with lymphangioleiomyomatosis.

LAM is a rare disorder that often occurs in patients with TSC (TSC-LAM). A rare sporadic form (S-LAM) affects the lungs, lymphatics, retroperitoneum, and kidneys^(4,12)^. TSC-LAM and S-LAM affect women almost exclusively and are characterized by diffuse interstitial proliferation of smooth muscle cell bundles and cystic changes in the pulmonary parenchyma. The hallmark feature of LAM is the presence of diffuse, well-circumscribed, thin-walled lung cysts distributed uniformly throughout the lungs^(4,12)^ (Figure 4). Clinically, LAM is characterized by progressive dyspnoea on exertion, recurrent pneumothoraces (Figure 5), and abdominal tumors, including AMLs and lymphangiomyomas^(4,12)^. Pneumothorax and chylous pleural effusion are the two major complications of pulmonary LAM^(4)^.

### Multifocal micronodular pneumocyte hyperplasia

Multifocal micronodular pneumocyte hyperplasia is the second lung manifestation in TSC. It is a rare pulmonary disorder characterized by multicentric, well-demarcated nodular proliferation of type II pneumocytes along alveolar septa^(5)^. Multifocal micronodular pneumocyte hyperplasia is characterized on high-resolution CT by multiple non-calcified pulmonary nodules or nodular ground-glass opacities ranging in size from 2 mm to 1 cm, scattered diffusely and randomly throughout the lung^(12)^.

## CARDIAC MANIFESTATIONS

Cardiac rhabdomyoma is a benign striated muscle tumor and the most common cardiac tumor in children^(4,5,13)^. It occurs in about 60% of children, but only 20% of adults, with TSC^(4,5,13,14)^. Most rhabdomyomas are asymptomatic, with a minority of patients experiencing arrhythmia and/or cardiac failure^(1,5)^. Echocardiography has been established as the primary diagnostic tool for the evaluation of cardiac rhabdomyomas in children^(3,4)^. CT or MRI can provide additional information regarding tumour extension or size, especially in older patients or those from whom echocardiographic images are poor due to bone or lung interference^(4)^.

## RENAL MANIFESTATIONS

### Renal angiomyolipoma

**Figure 6:**
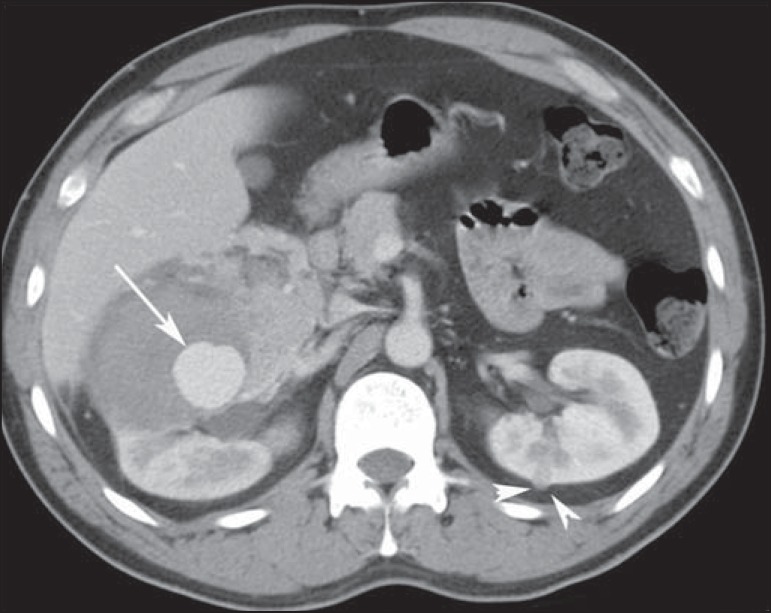
A 38-year-old man with tuberous sclerosis complex and renal lipid-poor angiomyolipoma. Axial contrast-enhanced CT image shows a large, high-attenuation, enhancing heterogeneous tumour with an aneurysm in the right kidney (arrow). This patient also had a small lipid-poor angiomyolipoma in the left kidney (arrowhead).

**Figure 7:**
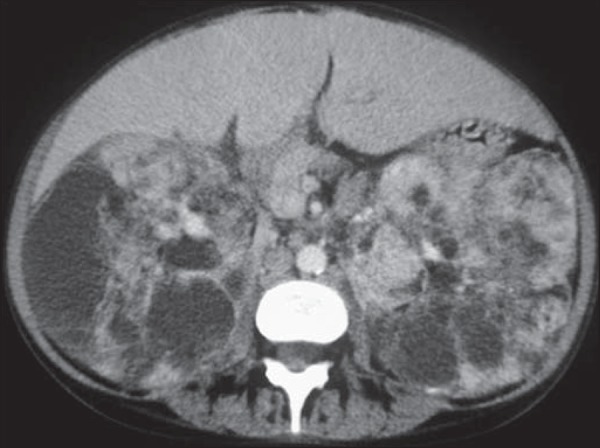
A 31-year-old woman with tuberous sclerosis complex and renal angiomyolipomas. Axial unenhanced CT image demonstrates multiple fat-containing tumors in the kidneys.

**Figure 8:**
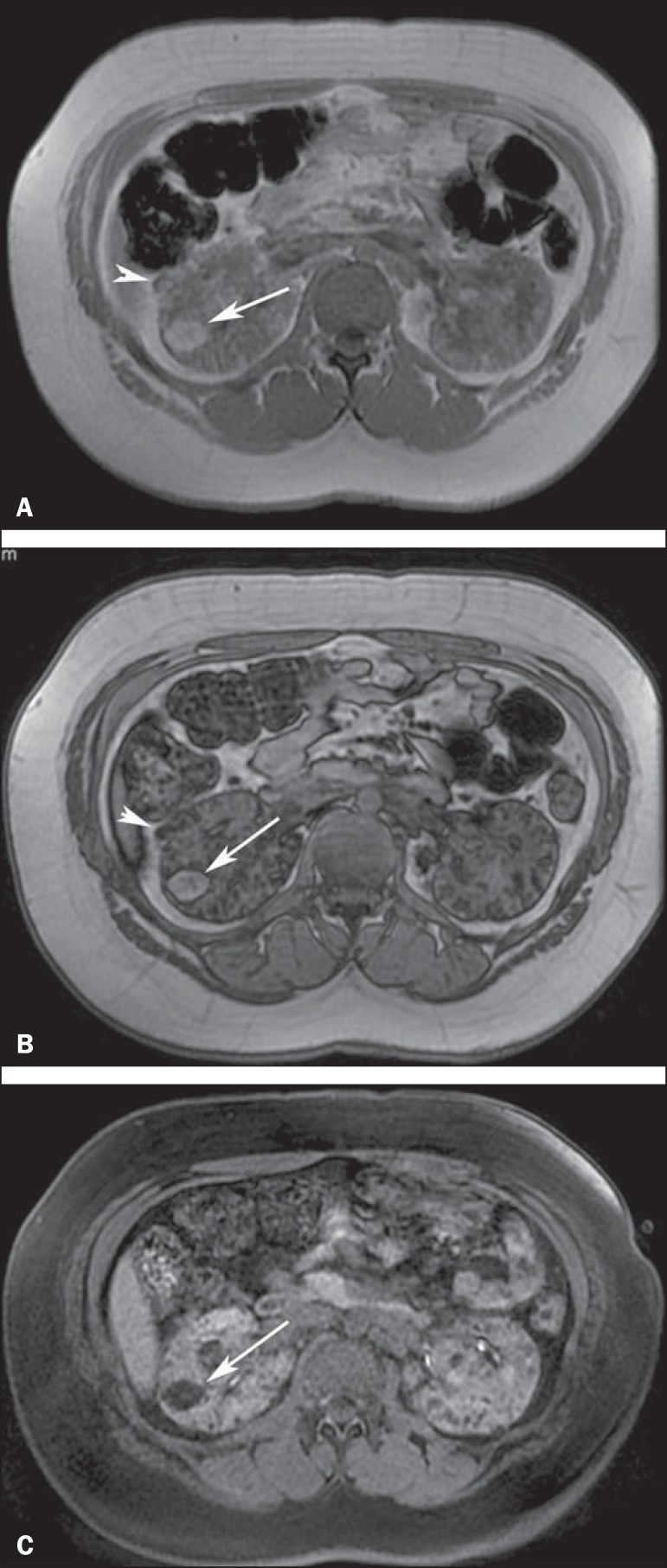
A 21-year-old woman with tuberous sclerosis complex and multiple renal angiomyolipomas. T1-weighted in-phase gradient-recalled echo image (**A**) shows a hyperintense right renal mass (arrow). T1-weighted opposed-phase gradient-recalled echo image (**B**) shows a peripheral India ink artefact at the fat-water interface between the mass and surrounding normal renal parenchyma, a finding diagnostic of lipid-rich angiomyolipoma. T1-weighted fat-suppressed image (**C**) shows diffuse intralesional low signal intensity in the lipid-rich angiomyolipoma. Note also a lipid-poor angiomyolipoma in the right kidney, appearing as a hyperintense nodular lesion in (**A**) (arrowhead) and presenting homogeneous internal signal loss in (**B**) (arrowhead) due to the presence of microscopic amounts of fat within the lesion.

AMLs are the most common mesenchymal renal neoplasms. They occur as isolated, sporadic entities in 80% of cases, most commonly manifesting in middle-aged women^(10)^. The other 20% of AMLs develop in association with TSC. AMLs are found in70-80% of patients with TSC^(4,5,13-15)^. The most alarming complication of renal AMLs is rupture, due to their abnormal vasculature, associated frequently with aneurysms^(2,13,14)^ (Figure 6). CT permits the diagnosis of renal AML by demonstrating the presence of intratumoral fat (Figure 7). Attenuation < -20 HU is a characteristic finding of AML in unenhanced CT examination^(4,15)^. AMLs with a predominant fatty component are isointense relative to fat on all MRI sequences^(3)^; their signal intensity is typically higher than that of the renal parenchyma on T1-weighted images, and homogeneous and high on T2-weighted images^(3,4)^ (Figure 8).

### Renal cell carcinoma

Renal cell carcinoma (RCC) is a rare manifestation in patients with TSC, with an estimated incidence of 2-4%, which is nonetheless higher than in the general population^(1,13,15,16)^. Imaging findings depend on RCC subtype. Clear cell RCC, the most common subtype, shows hypo- to isointensity on T1-weighted images and iso- to hyperintensity on T2-weighted images^(2-4)^. Papillary RCC commonly demonstrates low signal intensity on T2-weighted images and tends to exhibit hypovascular and homogeneous enhancement, which can be difficult to differentiate from that of lipid-poor AMLs^(4,15)^.

## OTHER MANIFESTATIONS

**Figure 9:**
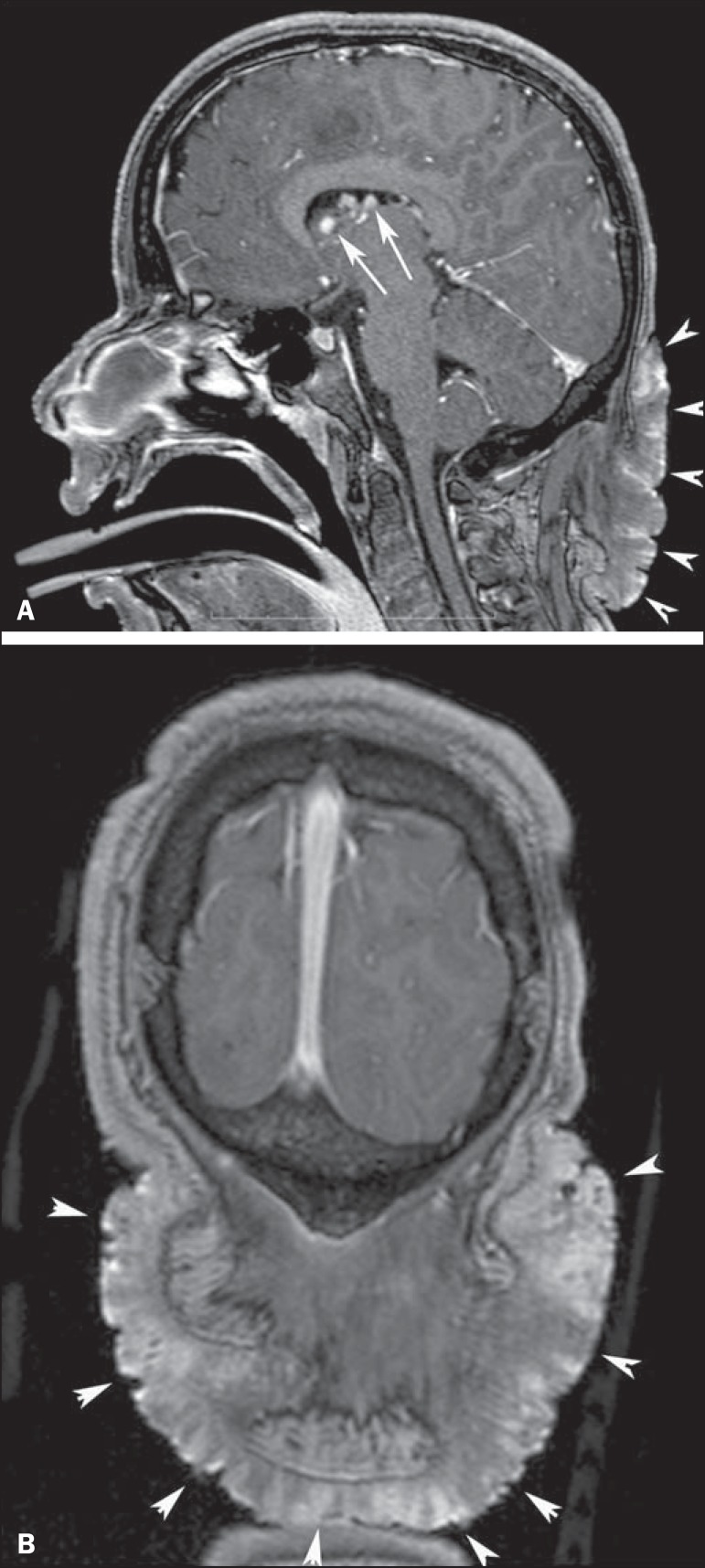
A 13-year-old boy with tuberous sclerosis complex. Sagittal (**A**) and coronal (**B**) T1-weighted images with gadolinium enhancement show a large, protuberant, irregularly thickened, elevated skin patch with an irregular border in the dorsal cervical region, consistent with shagreen patch (arrowheads). Note also in (**A**) enhancing subependymal nodules (arrows) projecting into the lumen of the lateral ventricle in the region of the foramen of Monro.

Detection of characteristic dermatological lesions, which can appear anytime during childhood, is important in the diagnosis of TSC. The most common lesions are hypomelanotic macules, angiofibromas, shagreen patches (Figure 9), forehead plaques, and ungual fibromas^(2,5)^.

**Figure 10:**
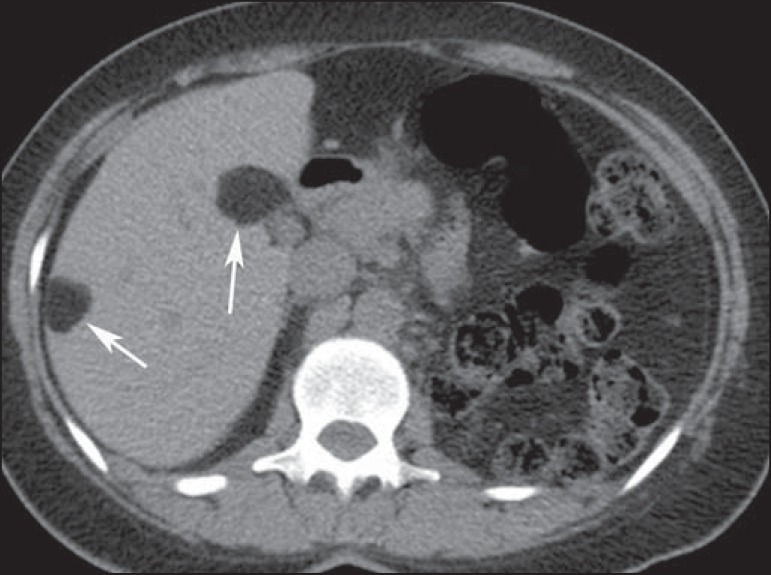
A 27-year-old man with tuberous sclerosis complex. Axial unenhanced CT image shows two well-defined nodular lesions with fat attenuation (arrows) in the liver, compatible with angiomyolipomas.

**Figure 11:**
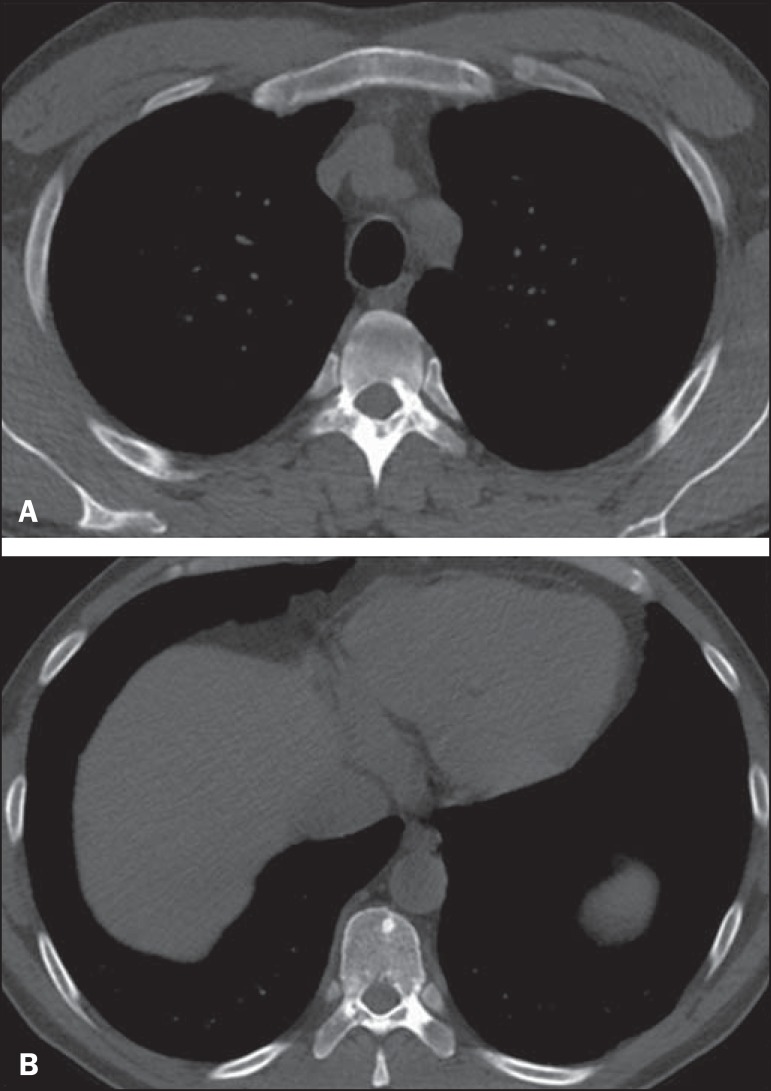
A 22-year-old woman with tuberous sclerosis complex and sclerotic spinal bone lesions. Axial CT images at the T5 (**A**) and T8 (**B**) levels demonstrate round, oval, and elongated sclerotic bone lesions on the anterior aspects of the vertebral bodies, pedicles, laminas, and transverse and spinous processes.

The involvement of various abdominal organs, including the alimentary tract, hepatobiliary system, and pancreas, has been reported in patients with TSC. Hepatobiliary lesions, including hepatomegaly, AMLs, lipomas, hamartomas, and fibromas, have been described in patients with TSC. Most hepatic AMLs are sporadic^(4)^. The incidence of hepatic AML (Figure 10) is much lower than that of renal AML in patients with TSC^(5)^. Skeletal abnormalities in TSC include cyst-like lesions, hyperostosis of the inner table of the calvaria, osteosclerotic changes (Figure 11), periosteal new bone formation, cystic changes in the phalanges, and scoliosis^(3)^. These bone abnormalities can also be seen in several other diaseases^(17-22)^, and only in the presence of changes in other organs that fulfill TSC criteria can be associated with the complex. Retinal hamartomas are present in about 40-50% of patients with TSC. When retinal hamartomas have calcification, ultrasound, CT, and MRI can show these lesions^(2)^.
